# Galectin 3 expression in regional lymph nodes and lymph node metastases of oral squamous cell carcinomas

**DOI:** 10.1186/s12885-018-4726-6

**Published:** 2018-08-16

**Authors:** Falk Wehrhan, Maike Büttner-Herold, Luitpold Distel, Jutta Ries, Patrick Moebius, Raimund Preidl, Carol I. Geppert, Friedrich W. Neukam, Marco Kesting, Manuel Weber

**Affiliations:** 1 Department of Oral and Maxillofacial Surgery, Friedrich-Alexander University Erlangen-Nürnberg, Glueckstrasse 11, 91054 Erlangen, Germany; 2 Department of Nephropathology, Institute of Pathology, Friedrich-Alexander University Erlangen-Nürnberg, Erlangen, Germany; 3 Department of Radiation Oncology, Friedrich-Alexander University Erlangen-Nürnberg, Erlangen, Germany; 4 Institute of Pathology, Friedrich-Alexander University Erlangen-Nürnberg, Erlangen, Germany

**Keywords:** Oral squamous cell carcinoma, Oscc, Lymph node, Metastasis, Oral cancer, Galectin 3, Gal3, Macrophage polarization, M1, M2, Peripheral tolerance, Immune checkpoint

## Abstract

**Background:**

Neck dissection is standard in surgical management of oral squamous cell carcinomas (oscc). However, the immunologic link between primary tumor and lymph nodes is insufficiently understood. Galectin 3 (Gal3) promotes M2 polarization of macrophages and contributes to immunosuppression. The current study analyzes the association between Gal3 expression in regional lymph nodes of oscc with histomorphologic parameters (T-, N-, L- Pn-stage, grading) of the primary tumor. Additionally, Gal3 expression is correlated with markers of macrophage polarization (M1 vs. M2).

**Methods:**

Preoperative diagnostic biopsies (*n* = 26), tumor resection specimens (*n* = 34), tumor-free lymph nodes (*n* = 28) and lymph node metastases (*n* = 10) of T1/T2 oscc patients were immunohistochemically analyzed for Gal3 and macrophage marker (CD68, CD11c, CD163 and MRC1) expression. The number of positive cells and the expression ratios were quantitatively assessed.

**Results:**

High Gal3 expression in tumor-free regional lymph nodes was significantly (*p* < 0.05) associated with increased tumor size. The epithelial compartment of lymph node metastases showed a significantly (*p* < 0.05) increased Gal3 expression compared to biopsies and tumor resection specimens. Cell density of M2 macrophages was significantly (*p* < 0.05) and positively correlated with the number of Gal3 expressing cells in lymph nodes and tumor specimens.

**Conclusion:**

Gal3 expression in regional lymph nodes might be associated with oscc progression. The increased Gal3 expression in regional lymph nodes of larger tumors underlines the need of immunomodulatory treatment concepts in early-stage oscc. Blocking of Gal3 might be a therapeutic option in oral cancer.

## Background

Removal of cervical lymph nodes is standard in surgical treatment of primary oral squamous cell carcinomas (oscc) even in the absence of radiologic signs of lymph node metastases [1, 2]. This approach is widely accepted and covered by major treatment guidelines [3, 4]. In neck dissection specimens of 20% to 40% of radiologically nodal negative (cN0) oscc cases, lymph node metastases are histologically detected [4]. Diagnostic value of neck dissection for prognostic assessment and planning of adjuvant radiotherapy has long been accepted [1]. However, the therapeutic value of elective neck dissection in cN0 cases was discussed controversially. A study by D’Cruz et al. was the first large prospective randomized trial showing the role of neck dissection as therapeutic procedure [5]. Elective neck dissection in cN0 oscc patients was shown to improve overall survival and disease-free survival compared to a wait and watch approach [5].

Besides this empiric evidence, there is little known regarding the biologic connection between the primary oral cancer and the regional lymph nodes. Especially the immunologic interaction of tumor and regional lymph nodes is still not sufficiently understood [6]. Since the 1970s and 1980s there is evidence that the primary tumor has an immunologic impact on the draining lymph nodes [7, 8]. Later it was shown that oral carcinomas control sinus formation and lymph vessel density in cervical lymphatic tissue [9, 10].

Regional lymph nodes are important for antigen presentation and the initiation of an adaptive immune response against tumor cells as antigen presenting cells recirculate from the primary tumor site to the lymph nodes and prime a specific T-cell response [11]. Macrophages are important antigen presenting cells and critical for an anti-tumor host reaction [12]. Especially, the polarization of macrophages (M1 vs. M2) is of importance in the context of malignant diseases [6, 13, 14]. M1-polarized macrophages are capable of efficient antigen presentation and T-cell activation, supporting tumor clearance, whereas M2 macrophages have immunosuppressive capabilities and are associated with tumor promotion [12, 15].

Galectin 3 (Gal3) is a phylogenetically highly conserved protein involved in embryologic development and immune regulation [16]. Gal3 is expressed by several immune cells including macrophages, dendritic cells and activated lymphocytes [16]. Gal3 was shown to shift macrophage polarization towards the immunosuppressive M2-type [17]. Additionally, Gal3 can inhibit the differentiation of monocytes into dendritic cells, which compromises their ability of antigen presentation and immune activation [17]. In oscc tumor tissue, high Gal3 expression was shown to correlate with histomorphologic parameters of malignancy. Accordingly, oscc with histologically proven lymph node metastases (pN+) showed a significantly increased infiltration of Gal3 positive cells in tumor resection specimens [18]. Additionally, larger tumor size (T2 vs. T1) was associated with higher Gal3 expression [18]. In contrast to primary tumor tissue, the regulation of Gal3 expression in regional lymph nodes of oscc patients has not yet been investigated.

However, it has been shown that macrophage polarization in tumor-free lymph node specimens, obtained during neck dissection surgery, was associated with parameters of malignancy of the primary tumor [6]. Oscc cases with high tumor grading and cases with increased T-, L- and Pn-status showed a shift of macrophage polarization in regional lymph nodes towards the tumor-promoting M2-type [6].

In the current study macrophage polarization markers were used that are frequently described in literature and were successfully applied in previous projects of our group [19–22]. CD68 is the most commonly used generic macrophage marker [23, 24]. CD11c is a frequently used M1 macrophage marker [6, 20, 24–27]. M2 macrophages can be identified by staining the CD163 and the MRC1 antigen [6, 20, 27–29].

We formulated the hypothesis Gal3 might contribute to local immunosuppression in oral cancer and that Gal3 expression is might correlate with M2 polarization of macrophages in tumor tissue and regional lymph nodes of oscc. To test this hypothesis, the current study aims to analyze if Gal3 expression in regional lymph nodes obtained during neck dissection surgery in oscc patients is associated with histomorphologic parameters of the primary tumor (T-, N-, L-, Pn-status, grading) and macrophage polarization. Therefore, Gal3 expression was correlated with immunohistochemical markers of macrophage polarization (CD68, CD11c, CD163, MRC1). Moreover, Gal3 expression in diagnostic biopsies, tumor resection specimens and lymph node metastases was compared.

## Methods

### Patients and tissue collection

In this retrospective study, samples from 34 patients with primary pT1 and pT2 OSCC were included. Biopsy specimens from 26 patients, tumor resection specimens from 34 patients, tumor-free cervical lymph nodes from 28 patients (23 lymph node specimens for Gal3 staining) and lymph node metastases from 10 patients from neck dissections were available and suitable for immunohistochemical analysis. Biopsy-, tumor resection- and cervical lymph node-specimens originate from the same patients. Biopsies of the included patients were analyzed additionally to tumor resection specimens as previous studies showed differences in the immune environment between biopsy- and tumor resection samples. The mean time between preoperative diagnostic incision biopsy and tumor resection was 15 days (SD 9.6). All patients were treated in 2011 at the Department of Oral and Maxillofacial Surgery of the University Hospital Erlangen. The study protocol was approved by the ethical committee of the University of Erlangen-Nuremberg (Ref.-No. 45_12 Bc). Tissue specimens collected for routine histopathologic diagnosis were used. All tissue samples were judged by a pathologist during routine pathological assessment. Histomorphologic tumor parameters (T-, N-, L-, Pn- status, grading) were obtained from routine pathology records. Patients with preoperative radio- or chemotherapy, with distant metastases and with other malignancies were excluded.

The patient cohort (*n* = 34) consisted of 11 patients with a tumor of the tongue, 11 patients with a tumor of the floor of the mouth, 8 with a tumor of the alveolar crest, 3 with a tumor of the palate and 1 with a tumor of the cheek.

The average age of the patients (23 males and 11 females) was 63 years. The N-status was N0 in 19 cases and N+ in 15 cases. Histologic grading was G1 in 2 cases, G2 in 26 cases and G3 in 6 cases.

### Immunohistochemical staining and quantitative analysis

The immunohistochemical staining procedure was performed as previously described [20, 27]. The following primary antibodies were used: anti-Galectin 3 (sc-20,157, clone H-160, Santa Cruz, Dallas, Texas, USA), anti-CD11c (ab52632, clone EP1347y, Abcam, Cambridge, UK), anti-CD68 (11,081,401, clone KP1, Dako, Hamburg, Germany), anti-CD163 (MAB1652, clone K20-T, Abnova, Taipei City, Taiwan) and anti-MRC1 (H00004360–1102, clone 5C11, Abnova). Human tonsil tissue was included as positive control in each series.

An analysis of the correlation of macrophage polarization markers (CD68, CD11c, CD163 and MRC1) with histomorphologic parameters in this patient cohort was already published [6, 20, 27].

All specimens were completely scanned and digitized using the method of “whole slide imaging”. The scanning procedure was performed in cooperation with the Institute of Pathology of the University of Erlangen-Nürnberg using a Pannoramic 250 Flash III Scanner (3D Histech, Budapest, Hungary) in 40× magnification mode. All samples were digitally analyzed (Case viewer, 3D Histech, Budapest, Hungary). HE-stained sections of all samples were examined to ensure that all samples contained representative lymphatic resp. oscc tissue.

For biopsies, tumor resection specimens and lymph node metastases, three visual fields showing the highest infiltration rate of positive cells were selected in each (hot spot analysis), making a complete area of 1.1 to 1.5 mm^2^ per specimen (Case viewer, 3D Histech, Budapest, Hungary).

Micrographs of the visual fields were imported into the Biomas analysis software (modular systems of applied biology, Erlangen, Germany) for cell counting. For specimens from the primary tumor or nodal metastases two regions of interest were defined in the visual fields using the Biomas software: the epithelial tumor compartment and the tumor stroma. In tumor-free lymph nodes three visual fields showing the highest infiltration rate of positive cells (hot spot analysis) in the interfollicular zone (IFZ) of the lymph nodes and three visual fields showing the highest infiltration rates in the lymph node sinus were selected and also imported into the Biomas software. For the visual fields including lymph nodes sinuses, analyses were restricted to the sinus excluding the perisinusoidal zone.

A quantitative analysis was performed to determine the number of Galectin 3- and CD68-, CD11c-, CD163- and MRC1-positive cells. Assessment of the cell density per mm^2^ was performed as previously described [19, 20].

### Statistical analysis

To quantify the immunohistochemical staining, the cell count was determined as the number of positive cells per mm^2^ of the specimen. Multiple measurements were pooled for each sample group prior to analysis. The results are expressed as the median, standard deviation (SD) and range. Box plot diagrams represent the median, the interquartile range, minimum (Min) and maximum (Max). Two-sided, adjusted *p*-values ≤0.05 generated by the ANOVA test were considered to be significant.

Correlations analysis was performed using the Pearson correlation test. Pearson correlation values and the adjusted *p*-values are given. Correlation diagrams indicate the R^2^ linear value.

The analyses were performed using SPSS 22 for Mac OS (IBM Inc., New York, USA).

## Results

### General morphologic considerations

**Fig. 1:**
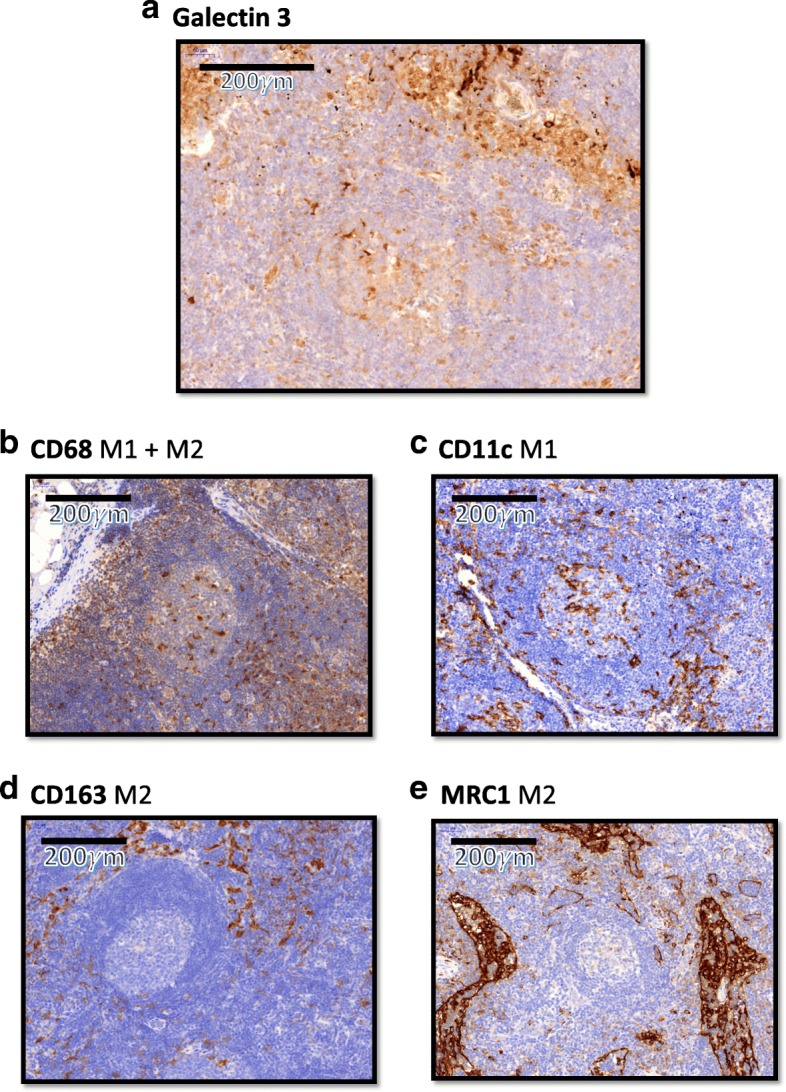
Distribution of Galectin 3 and macrophage marker expressing cells in lymph nodes. The figure displays the typical expression pattern of Galectin 3 positive cells (**a**) compared to the established macrophage markers CD68, CD11c, CD163 and MRC1. CD68 (**b**) is the best established pan-macrophage marker for M1- and M2-polarized macrophages. CD11c (**c**) is a marker for M1-polarized macrophages. CD163 (**d**) and MRC1 (**e**) are M2 macrophage markers. Corresponding virtual microscope images (20× magnification) of a tumor-free cervical lymph node of a oscc patient are shown

Galectin 3 (Gal3) expression was observed in all analyzed specimens. Gal3 showed a predominantly cytoplasmic expression pattern. In lymph nodes, most Gal3 expressing cells were found in the sinus (Fig. 1a). There was no accentuation of Gal3 expressing cells in the follicles. Gal3 positive cells showed a distribution pattern comparable to CD163 or MRC1 positive M2 macrophages as well as to the pan-macrophage marker CD68 (Fig. 1).

### Galectin 3 expression in tumor-free cervical lymph nodes of oscc patients

**Fig. 2:**
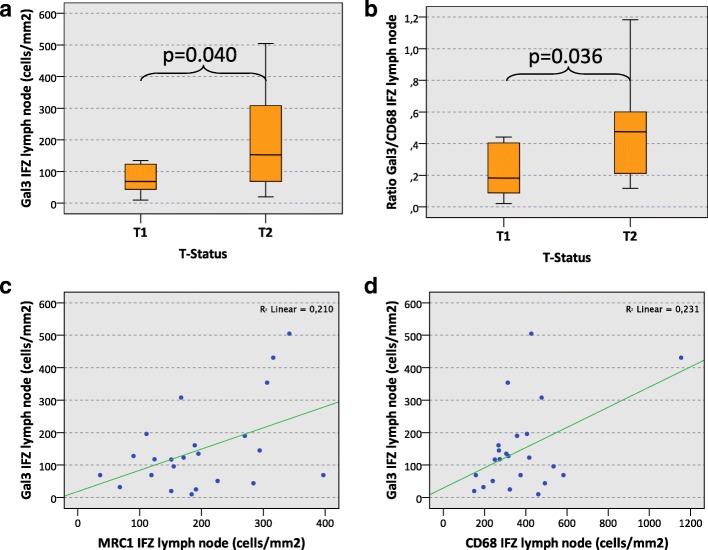
Galectin 3 expression in lymph nodes and correlation with macrophage markers. **a**) The figure shows Galectin 3 (Gal3) cell density (positive cells/mm^2^) in the interfollicular zone (IFZ) of tumor-free cervical lymph nodes of oscc patients depending on the T-Status (T1 vs. T2). *P*-value generated by the ANOVA test is indicated. **b**) The figure shows the Gal3/CD68 expression ratio in the interfollicular zone (IFZ) of tumor-free cervical lymph nodes of oscc patients depending on the T-status (T1 vs. T2). *P*-value generated by the ANOVA test is indicated. **c**) The scatter diagram shows the correlation of cell density (positive cells/mm^2^) of Gal3 and MRC1 expressing cells in the interfollicular zone (IFZ) of tumor-free cervical lymph nodes of oscc patients. The R^2^ linear value (Pearson correlation) is indicated. **d**) The scatter diagram shows the correlation of cell density (positive cells/mm^2^) of Gal3 and CD68 expressing cells in the interfollicular zone (IFZ) of tumor-free cervical lymph nodes of oscc patients. The R^2^ linear value (Pearson correlation) is indicated

In the interfollicular zone (IFZ) of tumor-free cervical lymph nodes of oscc patients, the Galectin 3 (Gal3) cell count in T2 oscc was significantly higher than in T1 cases (median 153 cells/mm^2^ and 69 cells/mm^2^, respectively, *p* = 0.040) (Table 1, Fig. 2a). In the lymph node sinus, there was no significant difference in Gal3 expression regarding the T-status (median T2 274 cells/mm^2^ and T1 175 cells/mm^2^; *p* = 0.357) (Table 1).

**Table 1:** Galectin 3 (Gal3) cell count (cells/mm^2^) and Gal3/CD68 expression ratio in lymph node specimens of oscc patients

*Gal3 expression in lymph nodes*									
Marker	*n*	Gal3 IFZ		Gal3 sinus		Ratio Gal3/CD68 IFZ		Ratio Gal3/CD68 sinus	
		Median	SD	Median	SD	Median	SD	Median	SD
*T-Status*									
*T1*	*9*	69	46	175	172	0.18	0.16	0.16	0.07
*T2*	*14*	153	152	274	272	0.48	0.33	0.34	0.21
*p*-value		**0.040**		0.357		**0.036**		**0.044**	
*N-Status*									
*N0*	*15*	96	144	209	235	0.37	0.29	0.21	0.23
*N+*	*8*	142	116	271	263	0.50	0.31	0.32	0.16
*p*-value		0.649		0.480		0.231		0.881	
*L-Status*									
*L0*	*17*	118	110	227	217	0.43	0.27	0.23	0.21
*L1*	*6*	176	186	277	334	0.27	0.40	0.31	0.18
*p*-value		0.289		0.412		0.816		0.865	
*Pn-Status*									
*Pn0*	*13*	123	122	196	241	0.41	0.31	0.23	0.22
*Pn1*	*7*	51	179	263	304	0.21	0.38	0.23	0.17
*p*-value		0.553		0.498		0.960		0.942	
*Grading*									
*G2*	*15*	118	124	249	224	0.42	0.32	0.26	0.20
*G3*	*6*	117	174	212	348	0.25	0.26	0.16	0.21
*p*-value		0.848		0.929		0.659		0.622	

Galectin 3 (Gal3) cell counts (positive cells/mm^2^) and the ratio of Galectin 3 and CD68 positive cells in cervical lymph nodes of oscc patients depending on histomorphologic parameters of the primary tumor (T-, N-, L-, Pn-Status, Grading). Results for the interfollicular zone (IFZ) and the sinuses of tumor-free lymph nodes are given. Values represent the median, standard deviation (SD) and *p*-value (ANOVA)

Significant *p*-values are indicated in bold letters

The ratio of Gal3-expressing cells and CD68-positive macrophages (Gal3/CD68-ratio) in the lymph node IFZ of T2 oscc cases was significantly higher compared to T1 cases (median value 0.48 and 0.18, respectively, *p* = 0.036) (Table 1, Fig. 2b). Assessing the Gal3/CD68-ratio in the lymph node sinus, T2 cases revealed a significantly higher ratio than T1 cases (median value 0.34 and 0.16, respectively, *p* = 0.044) (Table 1).

No significant association of Gal3 expression, Gal3/CD68 ratio, N-, L-, Pn-status and tumor grading in lymph node specimens was apparent.

There was no significant difference in Gal3 expression between the lymphatic compartment of tumor free lymph nodes and metastatic lymph nodes.

### Correlation of galectin 3 and macrophage marker expression in lymph nodes

In the interfollicular zone (IFZ) of tumor-free cervical lymph nodes, a significant positive correlation between Gal3 expression and CD68 expression was detectable (Pearson correlation + 0.480; *p* = 0.020) (Table 2, Fig. 2d). Additionally, a significant positive correlation between Gal3 expression and MRC1 expression in the IFZ was observed (Pearson correlation + 0.458; *p* = 0.028) (Table 2, Fig. 2c). Regarding macrophage marker expression in the IFZ, a significant positive correlation between CD68 and CD163 was evident (Pearson correlation + 0.449; *p* = 0.017) (Table 2).

**Table 2:** Correlation of Galectin 3 (Gal3) cell count (cells/mm^2^) in the IFZ of lymph nodes

		Gal3	CD68	CD11c	CD163	MRC1
Gal3	Pearson correlation	1				
	*p*-value					
	n	23				
CD68	Pearson correlation	**.480** ^*^	1			
	*p*-value	**0.020**				
	n	**23**	28			
CD11c	Pearson correlation	0.010	0.351	1		
	*p*-value	0.964	0.067			
	n	23	28	28		
CD163	Pearson correlation	−0.089	**.449** ^*^	0.100	1	
	*p*-value	0.687	**0.017**	0.612		
	n	23	**28**	28	28	
MRC1	Pearson correlation	**.458** ^*^	0.350	0.092	0.273	1
	*p*-value	**0.028**	0.068	0.640	0.160	
	n	**23**	28	28	28	28

The correlation of cell density (positive cells/mm^2^) of Galectin 3 (Gal3) positive cells and CD68, CD11c, CD163 and MRC1 expressing cells. Results for the interfollicular zone (IFZ) and the sinuses of tumor-free cervical lymph nodes of oscc patients are given. Values represent the Pearson correlation coefficient and *p*-value.

Significant correlations are marked with an * and printed in bold letters

### Correlation of galectin 3 and macrophage marker expression in tumor resection specimens

In the epithelial compartment of oscc tumor resection specimens a significant positive correlation between Gal3 and CD11c expression was observed (Pearson correlation + 0.385; *p* = 0.025) (Table 3).

**Table 3:** Correlation of Galectin 3 (Gal3) cell count (cells/mm^2^) in the epithelial compartment of tumor resection specimens

		Gal3	CD68	CD11c	CD163	MRC1
Gal3	Pearson correlation	1				
	*p*-value					
	n	34				
CD68	Pearson correlation	0.264	1			
	*p*-value	0.132				
	n	34	34			
CD11c	Pearson correlation	**.385** ^*^	**.641** ^*^	1		
	*p*-value	**0.025**	**< 0.001**			
	n	**34**	**34**	34		
CD163	Pearson correlation	0.035	**.643** ^*^	0.305	1	
	*p*-value	0.845	**< 0.001**	0.085		
	n	33	**33**	33	33	
MRC1	Pearson correlation	**.423** ^*^	**.749** ^*^	**.690** ^*^	**.563** ^*^	1
	*p*-value	**0.014**	**< 0.001**	**< 0.001**	**0.001**	
	n	**33**	**33**	**33**	**32**	33

The correlation of cell density (positive cells/mm^2^) of Galectin 3 (Gal3) positive cells and CD68, CD11c, CD163 and MRC1 expressing cells. Results for the epithelial compartment of oscc tumor resection specimens are given. Values represent the Pearson correlation coefficient and *p*-value.

Significant correlations are marked with an * and printed in bold letters

**Fig. 3:**
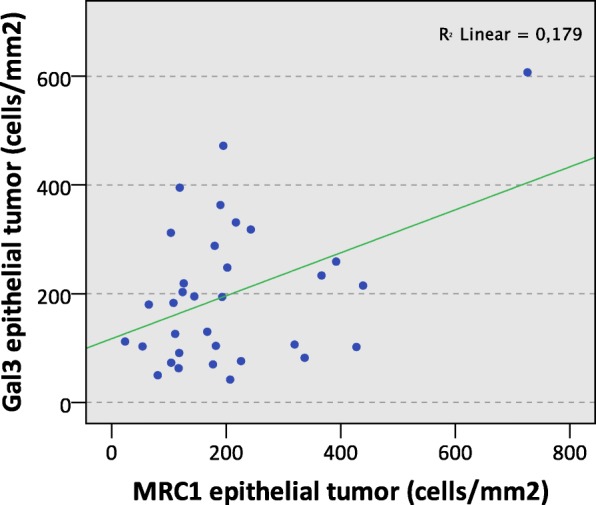
Correlation of Galectin 3 and MRC1 expression in oscc tumor specimens. The scatter diagram shows the correlation of cell density (positive cells/mm^2^) of Gal3 and MRC1 expressing cells in the epithelial compartment of oscc tumor resection specimens. The R^2^ linear value (Pearson correlation) is indicated

An even stronger positive correlation of Gal3 and MRC1 in the epithelial oscc compartment was detectable (Pearson correlation + 0.423; *p* = 0.014) (Table 3, Fig. 3).

Regarding macrophage marker expression, there was a highly significant positive correlation between CD68 expression and all other analyzed macrophage polarization markers CD11c, CD163 and MRC1 (CD68 vs. CD11c: Pearson correlation + 0.641; *p* < 0.001/CD68 vs. CD163: Pearson correlation + 0.643; *p* < 0.001/CD68 vs. MRC1: Pearson correlation + 0.749; *p* < 0.001) (Table 3).

Besides its positive correlation with Gal3, MRC1 expression also showed a highly significant positive correlation with macrophage polarization markers CD68, CD11c and CD163 (MRC1 vs. CD68: Pearson correlation + 0.749; *p* < 0.001/MRC1 vs. CD11c: Pearson correlation + 0.690; *p* < 0.001/MRC1 vs. CD163: Pearson correlation + 0.563; *p* = 0.001) (Table 3).

### Galectin 3 expression in oscc biopsy, tumor resection and lymph node metastasis specimens

**Fig. 4:**
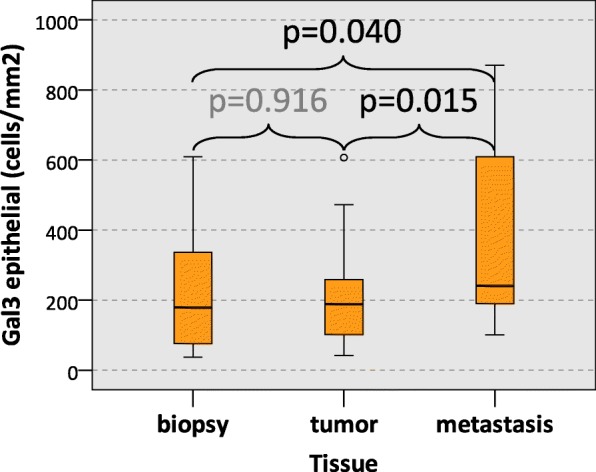
Galectin 3 expression in oscc biopsies, tumor resection specimens and metastases. The figure shows the Galectin 3 (Gal3) cell count (positive cells/mm^2^) in the epithelial compartment of diagnostic biopsies (biopsy), tumor resection specimens (tumor) and cervical lymph node metastases (metastasis) of oscc patients. *P*-values generated by the ANOVA test are indicated

A comparison of the Gal3 cell counts between oscc biopsies, tumor resection specimens and cervical lymph node metastases is given in Table 4. Analyzing the epithelial tumor compartment, lymph node metastases showed a significantly higher (median value 241 cells/mm^2^) Gal3 cell count than tumor resection specimens (median value 189 cells/mm^2^; *p* = 0.015) and diagnostic biopsies (median value 180 cells/mm^2^; *p* = 0.040) (Table 4, Fig. 4). The difference in Gal3 expression between oscc tumor resection specimens and biopsies was not statistically significant (Table 4, Fig. 4).

**Table 4:** Galectin 3 (Gal3) cell count (cells/mm^2^) in biopsies. Tumor resection specimens and lymph node metastases

*Gal3 cell count (cells/mm2)*							
Marker		Gal3 epithelial		Gal3 stromal		Gal3 epithelial + stromal	
		Median	SD	Median	SD	Median	SD
*Tissue*	*n*						
*biopsy*	*26*	180	159	189	175	196	154
*tumor*	*34*	189	130	254	259	212	177
*metastasis*	*10*	241	290	318	169	307	172
*p-values*							
biopsy vs. tumor		0.916		0.191		0.598	
biopsy vs. metastasis		**0.040**		0.655		0.178	
tumor vs. metastasis		**0.015**		0.931		0.485	

The Galectin 3 (Gal3) cell count (positive cells/mm^2^) in diagnostic biopsies (biopsy), tumor resection specimens (tumor) and lymph node metastases of oscc patients. Results for the epithelial tumor compartment (epithelial), the tumor stroma (stroma) and the whole analyzed area (epithelial + stroma) are given. Values represent the median, standard deviation (SD) and *p*-value (ANOVA)

Significant *p*-values are indicated in bold letters

In the tumor stroma and in the whole analyzed specimen area (epithelial + stroma) there was no statistically significant difference between biopsies, tumor resection samples and lymph node metastases evident (Table 4).

## Discussion

In the present study, Gal3 expression in tumor-free regional lymph nodes was associated with the tumor size of the primary oscc. Density of Gal3 positive cells in the interfollicular zone (IFZ) of lymph nodes of T2 tumors was significantly higher than in T1 carcinomas. Furthermore, the ratio of Gal3 expressing cells and CD68 positive macrophages was significantly higher in lymph nodes of T2 carcinomas. This effect could be observed in the IFZ and in the lymph node sinus. The increase of Gal3 might indicate an increase of immune tolerance in cervical lymph nodes associated with growing tumor mass.

Central and peripheral immune tolerance mechanisms can be differentiated. Gal3 is involved in negative selection of T-lymphocytes during thymic T-cell maturation [30] and thus contributes to central immune tolerance. Additionally, Gal3 is involved in peripheral immune tolerance. In this context, Gal3 can act as immune checkpoint interacting with lymphocyte activation gene (LAG)-3 or TIM-3 on T-cells leading to T-cell inactivation. Moreover, Gal3 can inhibit natural killer (NK) cell activation and cytokine production [31]. Thus, high Gal3 expression might impact the cellular immune response against cancer cells as a mean of immune evasion.

Immune checkpoints are gaining importance for prognostic evaluation, as well as therapeutic targeting of oral cancer. High expression of the immune checkpoint programmed cell death ligand 1 (PD-L1) in oscc specimens in immunohistochemistry correlated with the presence of lymph node metastases (N+) [32, 33]. Increased PD-L1 mRNA expression in peripheral blood of oral cancer patients was also shown to be associated with lymph node metastases [34]. These findings underline the role of peripheral immune tolerance for the progression of oscc. Therefore, Gal3 inhibition could be a potential therapeutic target in oral cancer, especially in combination with other checkpoint inhibitors.

In the current study, Gal3 expression in the epithelial compartment of lymph node metastases was significantly higher compared to specimens of the primary tumor site and preoperative biopsies. In contrast to the change in macrophage polarization in the time interval between diagnostic biopsy and tumor resection [20], there was no change in Gal3 expression observable. This indicates that Gal3 expression might be less susceptible to factors induced by the biopsy-derived tissue trauma than macrophage polarization, which shifts towards an M2-phenotype [20].

The increase of Gal3 expression in lymph node metastases might mediate an increased state of immune tolerance during metastatic progression of oral cancer. Besides oscc, a negative tumorbiological effect of Gal3 was shown in several other malignancies. Metastasized thyroid cancer showed increased Gal3 expression and knock-down of Gal3 in vitro resulted in reduced migration of thyroid cancer cells [35]. In colon cancer, high Gal3 expression was associated with larger tumor size, poor differentiation and poor overall survival [36].

As Gal3 might act as an immune checkpoint [31], the microenvironment of oscc lymph node metastases might inactivate tumor-infiltrating T-cells in a Gal3 dependent manner. Another possible mechanism, by which Gal3 might inactivate tumor specific T-cell responses was recently described [37]. Gal3 binds glycosylated proteins of the extracellular matrix (ECM) including laminin. Simultaneously, Gal3 can bind to glycosylated cytokines like IFNγ and thus reduce the diffusion of the cytokine through the extracellular matrix [37]. A decreased cytokine gradient reduces T-cell infiltration into the tumor. Transfer of T-cells in a mouse tumor model inhibited tumor growth only when Gal3 was simultaneously blocked [37]. Moreover, Gal3 contributes to secretion of a dense ECM and might thereby additionally block cytokine gradients and thus limit T-cell infiltration in the epithelial tumor compartment [37]. This is relevant for immune therapies, as checkpoint inhibitors, like PD1 blocking drugs, show the best efficiency in tumors with high T-cell infiltration rates [38].

The increased Gal3 expression in lymph node metastases suggests that metastases are less susceptible to the immune system than the primary tumor. Therefore, as long as adjuvant immune modulating treatment strategies are not available for early stage oral cancer, the elective surgical removal of cervical lymph nodes seems to be necessary to eliminate possible occult lymph node metastases.

An accentuation of Gal3 positive cells in the lymph node sinus was detected. The expression pattern of Gal3 is comparable to the M2 macrophage markers CD163 and MRC1 as well as to the pan-macrophage marker CD68. However, the M1 macrophage marker CD11c shows a different expression pattern. There is no prominence of CD11c expression in the lymph node sinuses. In contrast, the CD11c positive cells are distributed in the interfollicular zone (IFZ) and in the follicles.

Pearson correlation revealed, that Gal3 expressing cells show a significant positive correlation with CD68 positive macrophages and MRC1 positive M2 macrophages in the IFZ of tumor-free cervical lymph nodes. In tumor resection specimens, there was a significant positive correlation between Gal3 expressing cells and MRC1 positive macrophages. These data support the hypothesis that a relevant part of the Gal3 positive cells are M2-polarized macrophages [18]. M2-polarized macrophages might utilize Gal3 as immune checkpoint molecule to inhibit T-cell activation.

In tumor resection specimens, the pan-macrophage marker CD68 showed a significant positive correlation with the other investigated macrophage markers (CD11c, CD163 and MRC1). This finding indicates that the available macrophage markers include a relatively homogenous cell population in tumor specimens and are suitable for the immunohistochemical analysis of macrophage polarization. Although, all macrophage polarization markers showed a strong positive correlation in tumor resection specimens, an association between macrophage expression ratios with histomorphologic parameters could be proven [27]. This indicates that several macrophage markers should be analyzed simultaneously to describe macrophage polarization despite the fact that CD11c, CD163 and MRC1 expression correlates positively with CD68.

In lymph node specimens, macrophage markers showed a diminished positive correlation compared to tumor specimens. This suggests that macrophages in lymph nodes might be more heterogeneous than in tumors. Therefore, it might be necessary to separately analyze the different anatomic compartments of lymph nodes and to calculate macrophage expression ratios [6].

Oral cancer seems to have a complex influence on the regional lymphatic tissue. In addition to the proven association of oscc with macrophage polarization in draining lymph nodes [6] we could show a connection of the primary tumor to the immune regulatory Gal3 in lymph nodes. This underlines the need for consideration the regional and systemic immunologic status when investigating in tumor immunology.

As the current study was designed as a pilot study to analyze Gal3 expression in regional lymph nodes of oscc for the first time, the case number is relatively low.

There is evidence that nuclear vs. cytoplasmic expression of Gal3 might influence the biologic effect. Although the expression pattern of Gal3 was predominantly cytoplasmic in this analysis, a distinction between cytoplasmic and nuclear expression of Gal3 could not be performed.

The results of the current study show a positive correlation between Gal3 expressing cells and macrophages. However, the exact proportion macrophages that express Gal3 as well as the lineage of Gal3 expressing cells in lymph nodes and tumor tissue could not be determined by this analysis. In tumor specimens, besides macrophages, tumor cells can also express Gal 3 [18]. In lymph nodes, T-cells might contribute to Gal3 expression [18]. Finally, the value of the currently available macrophage polarization markers is not finally assessed [20].

## Conclusion

Besides the known association between high Gal3 expression in oscc tumor tissue and histomorphologic parameters of malignancy, Gal3 expression in regional lymph nodes might also be associated with oscc progression. This indicates a possible locoregional or systemical state of Gal3 mediated immunosuppression. A high infiltration of Gal3 positive cells is associated with M2 polarization of macrophages in tumor specimens and in regional lymph nodes. Blocking of Gal3 – potentially in combination with other immune checkpoint inhibitors – might be a therapeutic option in oral cancer. The fact that Gal3 expression in regional lymph nodes of T2 tumors is higher than in T1 tumors underlines the need of immunomodulatory treatment concepts in early-stage oral cancer.

## Abbreviations

ECM: extracellular matrix
Gal3: Galectin 3
IFZ: interfollicular zone
LAG-3: Lymphocyte-activation gene 3
M1: M1 polarized macrophages
M2: M2 polarized macrophages
oscc: Oral squamous cell carcinoma
PD-L1: programmed cell death ligand 1
PI3K: Phosphatidylinositol 3-kinase
SD: Standard deviation
TIM-3: T-cell immunoglobulin and mucin-domain containing-3
